# Improved Performance of D-Psicose 3-Epimerase by Immobilisation on Amino-Epoxide Support with Intense Multipoint Attachment

**DOI:** 10.3390/foods10040831

**Published:** 2021-04-11

**Authors:** Yifan Bu, Tao Zhang, Bo Jiang, Jingjing Chen

**Affiliations:** 1 State Key Laboratory of Food Science and Technology, Jiangnan University, Wuxi 214122, China; buyifan18@163.com (Y.B.); zhangtao@jiangnan.edu.cn (T.Z.); jingjinc@jiangnan.edu.cn (J.C.); 2 International Joint Laboratory on Food Safety, Jiangnan University, Wuxi 214122, China

**Keywords:** immobilisation, D-psicose 3-epimerase, amino-epoxide, D-allulose, HFA, glycine

## Abstract

D-allulose is an epimer of D-fructose at the C-3 position. With similar sweetness to sucrose and a low-calorie profile, D-allulose has been considered a promising functional sweetener. D-psicose 3-epimerase (DPEase; EC 5.1.3.30) catalyses the synthesis of D-allulose from D-fructose. Immobilised enzymes are becoming increasingly popular because of their better stability and reusability. However, immobilised DPEase generally exhibits less activity or poses difficulty in separation. This study aimed to obtain immobilised DPEase with high catalytic activity, stability, and ease of separation from the reaction solution. In this study, DPEase was immobilised on an amino-epoxide support, ReliZyme HFA403/M (HFA), in four steps (ion exchange, covalent binding, glutaraldehyde crosslinking, and blocking). Glycine-blocked (four-step immobilisation) and unblocked (three-step immobilisation) immobilised DPEase exhibited activities of 103.5 and 138.8 U/g support, respectively, but contained equal amounts of protein. After incubation at 60 °C for 2 h, the residual activity of free enzyme decreased to 12.5%, but the activities of unblocked and blocked DPEase remained at 40.9% and 52.3%, respectively. Immobilisation also altered the substrate specificity of the enzyme, catalysing L-sorbose to L-tagatose and D-tagatose to D-sorbose. Overall, the immobilised DPEase with intense multipoint attachment, especially glycine-blocked DPEase, showed better properties than the free form, providing a superior potential for D-allulose biosynthesis.

## 1. Introduction

D-allulose (also known as D-psicose) is a rare sugar with a molecular formula of C_6_H_12_O_6_. Its sweetness is almost 70% of that reported for sucrose and it is a low-energy monosaccharide, which suggests that it can be used as an ideal alternative to traditional sugar for individuals with diabetes and obesity [1]. Therefore, there has been a growing interest in the potential use of D-allulose in recent years. D-allulose naturally exists in sugarcane, wheat, and other plants in extremely small quantities [2,3]. The chemical synthesis of D-allulose is challenged by a substantial number of by-products and a complex purification method [4]. Enzymatic production of D-allulose from D-fructose using D-psicose 3-epimerase (DPEase; EC 5.1.3.30) has many advantages, such as a relatively simple purification process and high product concentration [5]. Furthermore, owing to the excellent advantages of immobilised enzymes (reusability, improved stability, low economic cost, modulated enzyme selectivity and specificity, and reduced inhibition) [6,7] compared to the free form, the preparation of D-allulose by immobilised DPEase has attracted considerable attention. DPEase has been reported to be immobilised on various carriers, but lower enzyme activity increases the cost [8,9,10,11,12]. Ran et al. [13] developed various polyhydroxyalkanoate nanoparticles decorated with DPEase in a recombinant endotoxin-free *clearcoli*, and the activity of the immobilised enzyme was as high as 649.3 U/g carrier. However, the particle size of the carrier was 50–200 nm and could only be separated by centrifugation, which limited its practical application. Therefore, for suitability for D-allulose synthesis, a better carrier and method must be developed for higher enzyme activity and stability.

Immobilisation of enzymes on epoxy supports is a good strategy as epoxy groups exhibit reaction with different groups (e.g., amino and thiol moieties) at the protein surface to form intense covalent bonds [14,15]. The chemical modification in this process exerts minimal effect on enzymes, for example, the pK value of the secondary amine group formed by the amino group of the protein and the epoxy group of the resin is similar to that of the amino group [14,15]. Nevertheless, under mild immobilisation conditions (low ionic strength and relatively neutral pH), the reaction activity of the epoxy group is reportedly unsatisfactory [16]. Hence, the immobilisation of enzymes on epoxy resin usually depends on a different mechanism. The first step is the physical adsorption of the protein on the carrier to facilitate the second step of covalent binding between the enzyme and epoxy groups of the resin. Under such conditions, several carriers have been designed to exhibit hydrophobicity. A high ionic strength is used to promote the hydrophobic adsorption of proteins during immobilisation. However, high ionic strength exerts a negative effect on certain enzymes and leads to the incurrence of high economic costs. The immobilisation rate of the enzyme on conventional monofunctional epoxy carriers is slow [14]. If the second group is introduced into the carrier to promote the physical adsorption of the enzyme (e.g., adsorption on immobilised metal chelates and ionic exchange), these conditions will improve considerably [14,15].

HFA, a heterofunctional-activated hydrophilic carrier, utilises amino-epoxide as its functional group [17]. Immobilisation involves two steps. First, the secondary amine on the resin dissociates hydroxide ions in solution and performs ion exchange with negatively charged enzyme proteins. This physical adsorption promotes the rapid accumulation of proteins on the resin surface, which enables an easy combination of the enzyme with epoxy groups. Second, epoxy groups exhibit reactions with different groups at the protein surface, generating a strong multipoint covalent attachment [14,18,19]. If there are excess epoxy groups present on the resin at the end of immobilisation, it may lead to overbinding with more groups of proteins during storage and use. The activity of the enzyme may decrease if the bound groups are located in the active centre of the enzyme [20]. As mentioned by Mateo et al. [14], to obtain better stability, amine and thiol compounds can be introduced on the epoxy support to prevent the binding of excess epoxy groups at the end of the immobilisation.

This study aimed to obtain immobilised DPEase with high catalytic activity, stability, and ease of separation. HFA is constituted by a polymethacrylate matrix with a particle size of 200–500 μm [17]. The relatively suitable particle size and the mechanical strength of HFA indicate that it can be easily separated from the reaction solution. DPEase was immobilised on HFA in four steps described as follows: (1) Ion exchange: Electrostatic attraction induced by negatively charged enzyme protein (pH greater than the isoelectric point) and positively charged support (containing secondary amine); (2) covalent binding: Epoxy groups of support and amino, sulfhydryl groups of enzyme; (3) crosslinking: Aldehyde group of glutaraldehyde and amino group of protein; and (4) blocking: The amino group of glycine and the remaining epoxy and aldehyde groups in the first three steps. After achieving the most suitable immobilisation conditions for each step, the enzymatic properties of the free enzyme, glycine-blocked enzyme, and unblocked enzyme were examined. To our knowledge, this is the first report on the immobilisation of DPEase on amino-epoxide support. This study aimed to obtain immobilised DPEase with remarkable multipoint attachment and to provide insights into the production of D-allulose.

## 2. Materials and Methods

### 2.1. Materials and Chemicals

Recombinant *Bacillus subtilis* 1A751/pUB-P43dpe-dal was constructed in reference to the method by reported He et al. [21] and the DPEase came from *Clostridium scindens* 35704. D-allulose (≥98.0% purity) was produced by our laboratory. D-fructose (≥99.0% purity) was purchased from Sangon Biotech Co., Ltd. (Shanghai, China). HFA was obtained from Resindion S.R.L (Mitsubishi Chem. Co., Milano, Italy).

### 2.2. Preparation of DPEase

To express DPEase, a single colony of recombinant *Bacillus subtilis* 1A751/pUB-P43dpe-dal was picked and cultured in Luria-Bertani (LB) medium and at 200 rpm, 37 °C for 12 h. An inoculum of 3% (*v*/*v*) was transferred into fresh fermentation medium and incubated at 37 °C until the highest DPEase activity was reached. Subsequently, the cultures were centrifuged at 13,000× *g* (12,000 rpm in NO. 6 rotor, TGL-20M centrifuge, Shanghai Lu Xiangyi Centrifuge Instrument Co., Ltd., Shanghai, China) at 4 °C for 15 min and the cell sediment was resuspended in sodium phosphate buffer (PB) (50 mM, pH 7.5). The suspended cells were disrupted by a high-pressure homogenizer (JHG-54-P100, Shanghai Precise Packaging Co., Ltd., Shanghai, China) at a pressure of 60 MPa for three consecutive times, followed by centrifugation at 13,000× *g* (12,000 rpm) for 15 min at 4 °C. The enzyme was purified by Ni^2+^-chelating Sepharose Fast Flow column. The order of loading was ultrapure water, binding buffer (50 mM PB, 500 mM NaCl, pH 7.5), crude enzyme solution, washing buffer (50 mM imidazole, 50 mM PB, 500 mM NaCl, pH 7.5), and elution buffer (500 mM imidazole, 50 mM PB, 500 mM NaCl, pH 7.5). Recombinant protein eluted in elution buffer was dialyzed for 72 h (4 °C, pH 7.5) in the order of PB (to remove imidazole), fresh PB containing ethylenediaminetetraacetic acid (EDTA) (to remove metal ion), and fresh PB (to remove EDTA). The purified enzyme was used for immobilisation.

### 2.3. Enzyme Activitity and Protein Concentration Assay

The enzyme reaction was performed by adding free enzyme solution or immobilised DPEase into the final volume of 1.0 mL PB (50 mM, pH 7.5) containing 90 g/L D-fructose and 1 mM Mn^2+^, then incubated at 55 °C for 10 min. The reaction was terminated by heating the mixture in boiling water for 5 min. After centrifugation at 12,100× *g* (13,400 rpm in NO. 2 rotor, MiniSpin centrifuge, Eppendorf AG, Hamburg, Germany; 4 °C, 10 min), the supernatant was further filtered for determination of D-allulose using high-performance liquid chromatography (HPLC) (e2695, Waters corp., Milford, MA, USA) equipped with a Ca^2+^ cation exchange column (Sugar-Pak 1, Waters corp., Milford, MA, USA). The column temperature was 85 °C and the mobile phase was ultrapure water with a flow rate of 0.4 mL/min. One unit of DPEase activity was defined as the amount of DPEase that catalysed the formation of 1 μmol of D-allulose per minute under assay conditions. The amount of D-allulose produced was quantified based on the peak area of the standard. Furthermore, the activity of free and immobilised enzyme was calculated using the following formulas:

Enzyme activity (U/mL) = *Q_allu_* × *V*_0_ × 1000/180/*t*/*V* (1)

Enzyme activity (U/g support) = *Q_allu_* × *V*_0_ × 1000/180/*t*/*m* (2)

where *Q_allu_* is the amount of D-allulose detected after reaction, mg/mL. *V*_0_ is the volume of reaction system, mL. *t* is the reaction time, min. *V* is the volume of free enzyme solution, and mL. *m* is the weight of immobilised enzyme, g.

The Bradford method [22] was used to determine the protein concentration. Coomassie Brilliant Blue-G 250 with 100 mg was dissolved in 50 mL ethanol (90%), then 100 mL of 85% phosphoric acid was added, and the volume was adjusted to 1 L with ultrapure water. The above-mixed liquid was defined as Reagent A. Add 5 mL of Reagent A to 1 mL of protein solution diluted to an appropriate concentration. After reacting at 25 °C for 20 min, the absorbance was measured at 595 nm (UV-1800, Aoyi Instruments Shanghai Co., Ltd., Shanghai, China). Additionally, bovine serum albumin was used as standard.

### 2.4. Immobilisation

#### 2.4.1. Immobilisation Method

Before immobilisation, amino-epoxide resin HFA was pretreated by soaking in a 4-fold volume of pure water for 30 min to remove dust and impurities and then washed with 50 mM PB (pH 7.5) to stabilize the pH. The immobilisation was then started with the addition of DPEase (20 h, 20 °C, 50 mM PB, pH 7.5). Subsequently, glutaraldehyde with final concentration of 0.01% was introduced to the mixture. After stirring for 1 h at 20 °C, the immobilised DPEase was rinsed 5 times with 50 mM PB (pH 7.5) to remove the unbound DPEase and residual glutaraldehyde. To block the rest of epoxy groups and aldehyde groups, the immobilised DPEase was incubated with 4-fold volume of 3 M glycine for 16 h at 20 °C. Finally, the immobilised protein was washed several times to remove the remaining glycine. The residual enzyme supernatants and washing solutions were collected to determine for enzyme activity and protein concentration. The immobilised DPEase were analysed for enzyme activity.

The activity recovery and protein loading efficiency were calculated using the following formulas:

Activity recovery (%) = (*U_imm_*/*U_applied_*) × 100 (3)

Protein loading efficiency (%) = [*(P_applied_* − *P_supernatant_* − *P_washing_)*/*P_applied_*] × 100 (4)

where *U_imm_* is the enzyme activity of immobilised DPEase and *U_applied_* is the activity of free enzyme added at the beginning of immobilisation. *P_applied_* is the amount of protein added, *P_supernatant_* is the residual activity in the supernatant after immobilisation, and *P_washing_* is the non-covalently bound protein observed in the washing solutions.

#### 2.4.2. Effect of Immobilisation Conditions on Enzyme Activities and Protein Loading Efficiency

The influence of conditions including enzyme loads (30–960 U of enzyme/g of HFA), time (in the range 0.5–53 h), temperature (in the range 4–50 °C), pH (in the range 6–10), and concentration of glutaraldehyde (in the range 0.005–0.1%) on enzyme immobilisation were determined.

### 2.5. Characterization of Immobilised DPEase

The detailed structure of immobilised DPEase (glycine-blocked, unblocked) was analysed on a scanning electron microscopy (SEM) (SU8100, Hitachi Ltd., Tokyo, Japan). The freeze-dried samples were spotted in the conductive glue with a tweezer and then sprayed with gold. The image of surface structure was obtained at an acceleration voltage of 3 kV.

The chemical composition and functional groups of immobilised DPEase was analysed on a Fourier-transform infrared spectroscopy (FT-IR) (IS10, Nicolet Instrument Corp., Waltham, MA, USA). Samples were mixed with KBr at the ratio of 1:80 and grounded into superfine powder. The powder was pressed into pellets and analysed on a FT-IR spectrophotometer, and the spectrum was collected over the range of 400–4000 cm^−1^.

### 2.6. Properties of Free and Immobilised DPEase

#### 2.6.1. Optimum pH and Temperature

The optimum pH of free and immobilised DPEase were determined by incubating the samples with D-fructose dissolved in the following buffers (50 mM each): PB (pH 6.0–7.5), Tris-HCl (pH 7.0–9.0), and glycine-NaOH (pH 8.5–9.5). The enzyme reaction was carried out at 55 °C for 10 min. The relative activity was expressed as the ratio of the enzyme activity measured at different pH values to the maximum enzyme activity.

Similarly, the optimum temperature of free and immobilised DPEase was evaluated at 35–70 °C at the corresponding optimal pH for 10 min. Maximal enzyme activity was defined as 100% relative activity.

#### 2.6.2. pH and thermal stability

To determine the pH and thermal stability, the DPEase derivatives were pre-incubated in buffers at pH from 6.0 to 10.0 (20 °C), or pre-incubated at 60 °C and 65 °C in 50 mM PB (pH 7.5). Samples were withdrawn at 1, 2, and 4 h, and the residual activity was measured under assay conditions. The initial enzyme activity was defined as 100%.

#### 2.6.3. Substrate Specificity

DPEase (enzyme dosage 75 U/g substrate) was added to four 90 g/L substrate solution (D-tagatose, L-sorbose, D-allulose, and D-fructose). Sugars and the mixture of sugar and resin were used as control groups. Samples were withdrawn after 24 h under the measuring conditions. After enzyme inactivation, the composition of the supernatant was analysed to determine the catalytic ability of DPEase on different substrates.

#### 2.6.4. Reusability

The reusability of immobilised DPEase was determined by repeated reactions. At the end of each cycle of reaction, the supernatant was withdrawn to determine the content of components. The immobilised enzyme was washed with PB (50 mM, pH 7.5) and added to fresh substrate solution for the next batch. The enzyme activity measured in the first round was defined as 100%.

### 2.7. Statistical Analysis

All experimental results were obtained by technical triplicates, and the results were expressed as the mean ± standard deviation. SPSS version 16.0 (SPSS, Chicago, IL, USA) was used for statistical analysis and the data were analysed by the Duncan test using one-way ANOVA. *p* < 0.05 was regarded as statistically significant.

## 3. Results and Discussion

### 3.1. Effect of Conditions on Immobilisation

**Figure 1:**
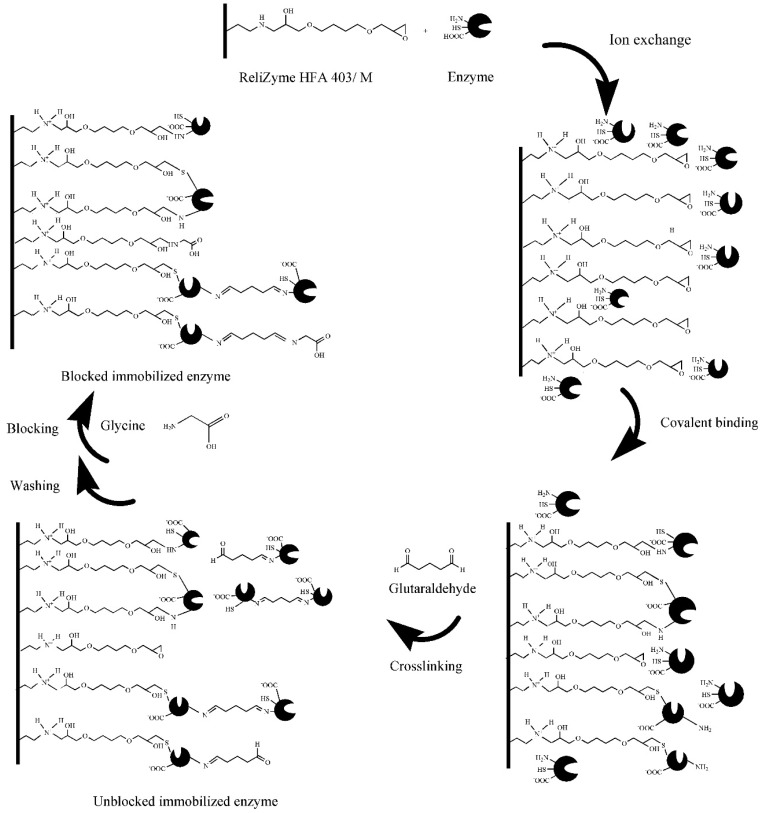
Schematic representation of enzyme immobilisation on HFA.

DPEase was immobilised in four steps on amino-epoxide resin HFA (Figure 1), which contains secondary amines that dissociate the hydroxyl ions into water and show weak alkalinity. When the pH of the solution was greater than the isoelectric point (PI) of the enzyme protein (PI ≈ 5.0), the enzyme protein was considered to be negatively charged. Under the conditions of ion exchange, proteins can be rapidly adsorbed onto the surface of the carrier. The epoxy groups of HFA may immediately undergo a ring-opening covalent binding reaction with groups of proteins to form secondary amino, thioether, and ester bonds. In the third step, glutaraldehyde was introduced to facilitate crosslinking with more enzyme proteins. After conducting washing steps, the introduction of glycine caused a reaction between its amino group and the excess epoxy and aldehyde groups.

**Figure 2:**
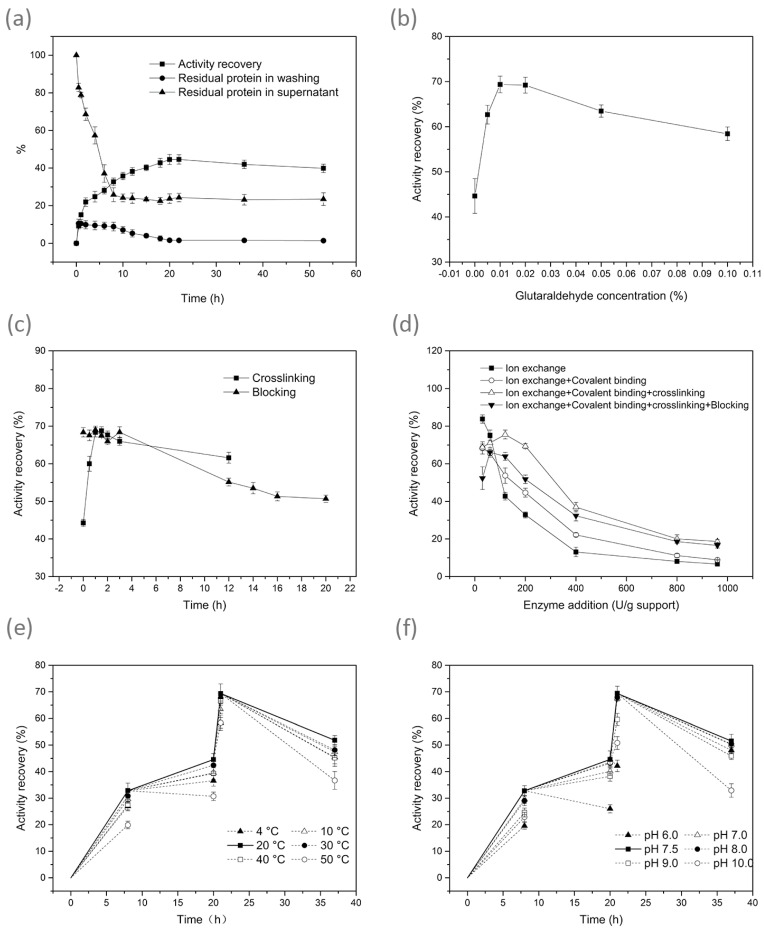
Effect of conditions on immobilisation. (**a**) Time of ion exchange and covalent binding; (**b**) concentration of glutaraldehyde; (**c**) time of crosslinking and blocking; (**d**) enzyme dosage; (**e**) temperature; 0–8 h: Ion exchange; 8–20 h: Covalent binding; 20–21 h: Crosslinking; 21–37 h: Blocking; (**f**) pH; 0–8 h: Ion exchange; 8–20 h: Covalent binding; 20–21 h: Crosslinking; 21–37 h: Blocking.

The effects of different conditions in each step are shown in Figure 2. As shown in Figure 2a, DPEase was immobilised on HFA for 53 h (20 °C, pH 7.5, enzyme dosage 200 U/g support). The residual protein content in the supernatant, washing solutions, and the activity of immobilised DPEase were determined at different intervals. Immobilisation of enzymes on amino-epoxide support usually requires a long duration for the completion of covalent binding [18]. In the present study, the protein content in the supernatant decreased rapidly in the first 8 h and remained almost unchanged thereafter. Although equal amounts of residual protein (*p >* 0.05) were determined in the supernatant at mid (8–20 h) and late stages (20–53 h) of immobilisation, relatively more protein content was lost due to leakage from enzyme supports that were immobilised for 8–20 h after performing rinsing step (*p* < 0.05). This suggests that protein immobilisation on amino-epoxide resin possibly involves the following two steps: The rapid adsorption of the enzyme onto the surface of the resin, and the slow reactions of the epoxy groups with the protein groups that resulted in the formation of covalent linkages for a long duration [18]. In other words, the proteins rinsed into the washing solutions were those that had not been bound or those with a weak bond formation. Therefore, an immobilisation time of 20 h is suitable for the covalent bond formation. Glutaraldehyde was used to enable crosslinking of more enzymes and to increase the intramolecular crosslinking. Crosslinking of subunits in the multimeric enzyme maintained the subunits together and stabilised the interaction between subunits [23]. Glutaraldehyde was introduced after DPEase was immobilised on the carrier for 20 h (20 °C, pH 7.5, and enzyme dosage 200 U/g support). Immobilisation for 20 h was defined as 0 h of crosslinking. The effect of the concentration and the time of crosslinking with glutaraldehyde on immobilisation were determined (Figure 2b,c). With the extension of the concentration and crosslinking time, the enzyme activity showed a trend of increase first and then a trend of decrease, reaching a peak at 0.01% in 1 h, respectively. Excessive crosslinking could lead to the distortion of the enzyme structure and reduced substrate accessibility [24]. Therefore, a decrease in enzyme activity was observed in later stages. After the crosslinked immobilised enzyme was subjected to washing steps, 3 M glycine was added to remove excess aldehyde and epoxy groups (20 °C, pH 7.5). Immobilisation for 21 h was defined as 0 h of blocking. The enzyme activity decreased slowly with incubation time and reached an equilibrium at 16 h (Figure 2c). Based on the above-mentioned considerations, the results for the best duration of each step are as follows: 0–8 h for ion exchange, 8–20 h for covalent binding, 20–21 h for crosslinking, and 21–37 h for blocking.

In terms of the amount of enzyme, proper dosage is vital for immobilisation on the amino-epoxide support. At 20 °C and pH 7.5, the effect of enzyme dosage exerted on the four steps was studied. Upon addition of 0–200 U/g support enzyme, the activity of immobilised DPEase increased rapidly. When the enzyme dosage was greater than 200 U/g support, the activity increased slowly and remained constant (Figure S1). As shown in Figure 2d, at lower enzyme dosages, the activity recovery of the immobilised enzyme in the latter three treatments was lower than that observed with the ion exchange step (*p* < 0.05). The addition of a small amount of enzyme could cause the high-density groups on the carrier to exhibit reactions with the groups present on different parts of the enzyme, which would destroy the active site and cause the dissociation of the polymer [25]. Glycine exerted a relatively less remarkable effect on enzyme activities at high enzyme dosages (*p >* 0.05). This may be attributable to the fact that epoxy groups had completely reacted with enzyme at high additions. Glycine was completely removed with the conduct of washing steps, and the lack of glycine did not result in the occurrence of greater steric hindrance. Considering both cases, an enzyme dosage of 200 U/g support was selected for the conduction of subsequent experiments.

The effect of temperature exerted on each step of immobilisation was studied under an enzyme dosage of 200 U/g support and pH 7.5. The immobilised enzymes all achieved the highest activity at 20 °C, as shown in Figure 2e. The different degrees of enzyme activity obtained at different temperatures may be attributed to enzyme inactivation, desorption, and the reactivity of different groups to temperature.

As illustrated in Figure 2f, immobilised DPEase achieved the highest activity recovery under conditions of pH 7.5 (enzyme dosage of 200 U/g support at 20 °C). Notably, the activity recovery at several pH values was significantly lower than that observed at other pH values (*p* < 0.05). Epoxy groups react with sulfhydril groups and carboxyl groups in acidic, neutral, and alkaline conditions, while with amino groups in neutral and alkaline conditions [26,27]. The mechanism of glutaraldehyde crosslinking is presently unclear; however, glutaraldehyde has been widely used in enzyme immobilisation as an excellent crosslinker. Enzyme protein groups, such as thiols and amines, can exhibit reactions with glutaraldehyde [24]. Okuda et al. [28] reported that thiol groups could exhibit reaction with glutaraldehyde only in the presence of a primary amino group. The reaction between glutaraldehyde and amino groups is relatively irreversible at pH 7.0–9.0, but it is reversible under acidic conditions [27]. However, apart from the above-mentioned points, it is equally important to consider the pH values at which the enzyme is relatively stable and would not show excessive covalent binding. Considering the above-mentioned aspects, a value of 7.5 was selected as the optimal pH for the conduction of each step.

The results of the four immobilisation steps are briefly shown in Table 1. The activity recoveries of the four immobilised DPEase were 32.8%, 44.6%, 69.4%, and 51.8%, respectively. The highest enzyme activity was observed in the latter two groups and blocked immobilised enzyme was lower than that of the unblocked enzyme. However, both demonstrated identical protein loading efficiencies of up to 90.6%. These results showed that the blocked and unblocked immobilised enzymes contained equal amounts of enzyme; however, the addition of glycine further affected the expression of enzyme activity. As reported by Konst et al. [20], after the performance of blocking with glycine, the activity recovery was reduced by 17%. This phenomenon may be explained by the occurrence of steric hindrance to substrate binding. Upon subjection to linkage with excess epoxy groups, the presence of glycine enabled the placement of the enzyme in close proximity and influenced activity expression. Even so, activities of 138.8 and 103.5 U/g in support of the unblocked and blocked immobilised DPEase were higher than those reported in the majority of studies, such as DPEase immobilised on *Bacillus subtilis* spores (4.5 U/g support) [12], on chitopearl beads BCW 2503 (5.2 U/g suport) [8], on ion exchange resin D301 (24.1 U/g support) [11], on ion exchange resin Duolite A568 (10.0 U/g support) [10], and on chitopearl beads BCW 2510 (63.0 U/g suport) [9].

In terms of activity recovery and protein loading efficiency, the latter two treatments (unblocked and blocked) may be the most desirable strategy to prepare immobilised DPEase for the synthesis of D-allulose.

### 3.2. Characterisation of Immobilised DPEase

**Figure 3:**
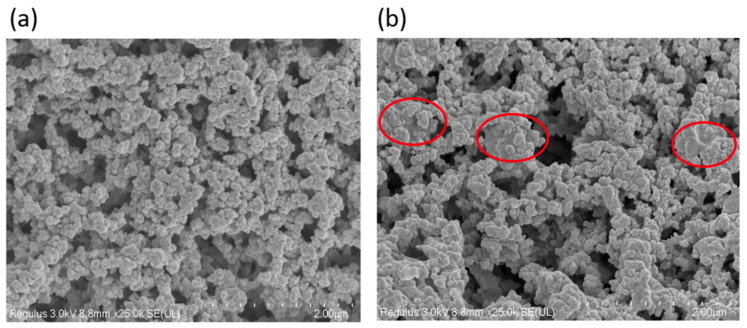
SEM images of the detailed surface structure of (**a**) unblocked and (**b**) blocked immobilised DPEase.

The detailed surface structure of the resin after immobilisation was observed by SEM (Figure 3). The images of immobilised DPEase (unblocked, blocked) showed that the pores of the resin were mostly filled with enzymes. Several mushy and uneven agglomerates (circle marked in Figure 3b) were observed on the immobilised DPEase blocked by glycine. Such substances may hinder the binding of the substrate to the enzyme and may cause discharge of products from the holes.

**Figure 4:**
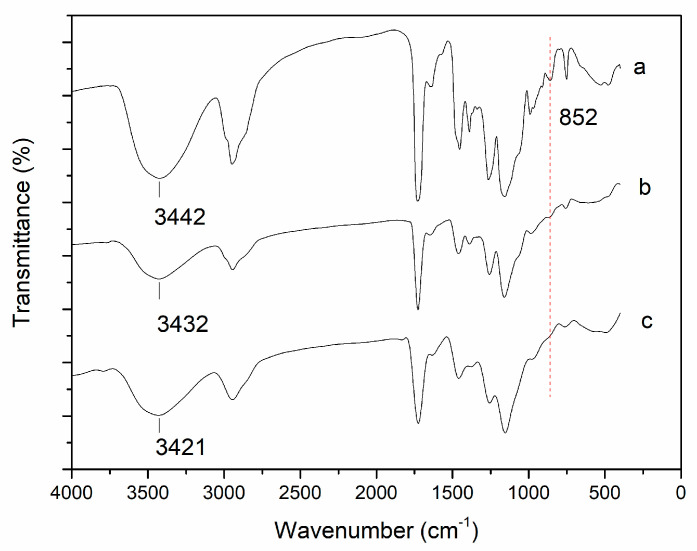
FT-IR spectra of the (**a**) native HFA, (**b**) unblocked DPEase, and (**c**) blocked DPEase.

The FT-IR image (Figure 4) shows the chemical composition and functional groups of each sample. The peak at 852 cm^–1^ denoted the characteristic absorption of the epoxy group and no peak of epoxy group was observed in the immobilised enzyme blocked by glycine. Moreover, the O-H bond wavenumbers of the native resin, unblocked, and blocked DPEase were 3442 cm^−1^, 3432 cm^−1^, and 3421 cm^−1^, respectively, indicating that epoxy groups bound to protein residues and enabled opening of the ring to generate alcoholic hydroxyl groups. These results indicated that the remaining epoxy groups were effectively blocked by glycine.

### 3.3. Optimum pH and Temperature of Free and Immobilised DPEase

**Figure 5:**
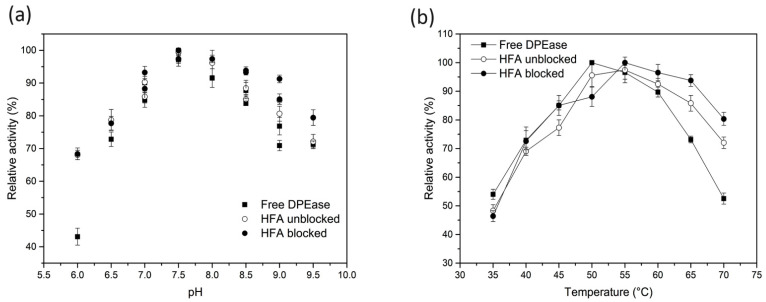
Optimum (**a**) pH and (**b**) temperature of free, unblocked, and blocked DPEase.

Environmental pH can alter the conformation of enzymes and may influence enzyme activity. The samples were equilibrated with their corresponding buffer solutions before determination. As shown in Figure 5a, all free and immobilised DPEase showed that the maximum activity occurred at pH 7.5. A shift in the optimum pH was not observed in this study, and a similar phenomenon was observed in other studies using epoxy resin [17,29]. Nevertheless, it should be noted that immobilised DPEase showed higher relative activity than free enzymes under acidic conditions (*p* < 0.05). This may be because the carrier was positively charged and attracted the negative charge in the solution, and a slightly alkaline microenvironment was then formed on the surface of the carrier.

As temperature exerts effects on enzyme activity, free and immobilised DPEase exhibited different trends (Figure 5b). The optimum reaction temperature of free DPEase was 50 °C, which was 5 °C lower than that of immobilised enzyme. The activity of unblocked immobilised DPEase decreased significantly (*p* < 0.05) when the reaction temperature was above 55 °C. This may be attributable to the excessive covalent binding of the enzyme to the residual epoxy groups. However, glycine can exhibit reactions with excessive epoxy groups and provide a more hydrophilic microenvironment [30,31]. The increase in the optimum reaction temperature is generally favourable, as it increases the reaction rate, decreases the viscosity, weakens the diffusion effect, and increases enzyme activity [32].

### 3.4. pH and Thermal Stability of Free And immobilised DPEase

**Figure 6:**
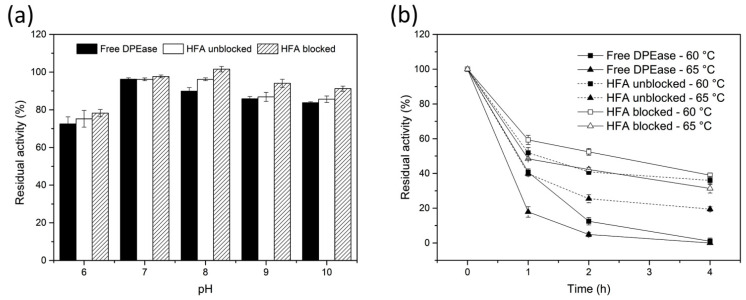
(**a**) pH stability and (**b**) thermostability of free and immobilised DPEase.

The pH stabilities of free and immobilised DPEase were determined at different pH values, and representative results are shown in Figure 6a. DPEase was relatively stable under both neutral and alkaline conditions. Under alkaline conditions, the stability of the immobilised enzyme without glycine blocking was lower than that of the blocked enzyme (p < 0.05). This may be because a certain duration of alkaline incubation improved the reactivity of epoxy groups with several groups and then led to the excessive formation or enhancement of the covalent bond between the enzyme and carrier [16,18]. This resulted in the destruction of the active site of the enzyme.

The increase in enzyme thermostability is helpful for potential industrial applications, such as reducing the risk of microbial contamination, increasing the solubility of the substrate, and accelerating the catalytic speed [29]. As shown in Figure 6b, the thermal stability of the immobilised enzyme was higher than that of the free enzyme. After immobilisation, the degree of conformational change in unfavourable environments decreased [33]. The carrier exerted a protective and shielding effect on the enzyme molecules, decreasing the sensitivity of the enzyme molecules to heat. Furthermore, multi-point covalent binding could reduce the unfolding and denaturation caused by thermal vibration between subunits [32].

### 3.5. Substrate Specificity

**Figure 7:**
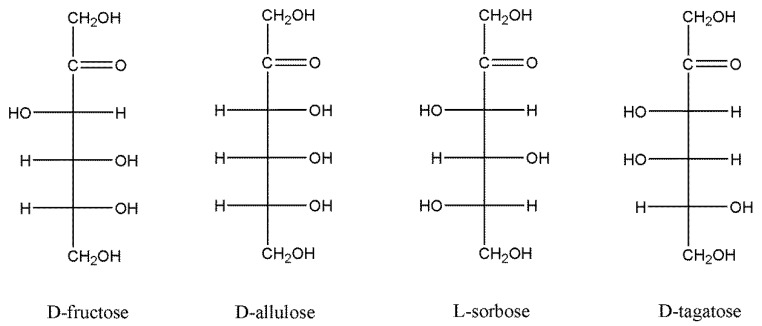
Fischer projection formulas of D-fructose, D-allulose, L-sorbose, and D-tagatose.

**Figure 8:**
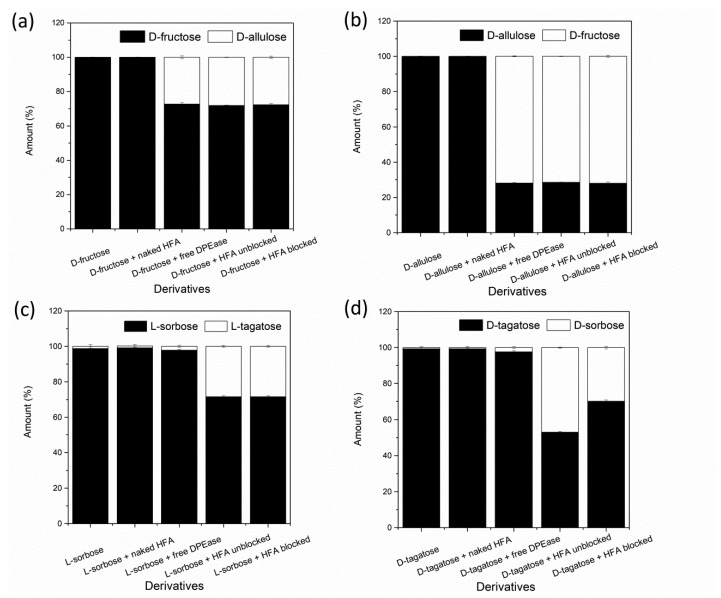
Substrate specificity of free and immobilised DPEase. (**a**) D-fructose/D-fructose + naked HFA/ D-fructose + free DPEase/D-fructose + HFA unblocked/D-fructose + HFA blocked DPEase; (**b**) D-allulose/D-allulose + naked HFA/D-allulose + free DPEase/D-allulose + HFA unblocked/D-allulose + HFA blocked; (**c**) L-sorbose/L-sorbose + naked HFA/L-sorbose + free DPEase/L-sorbose + HFA unblocked/L-sorbose + HFA blocked; (**d**) D-tagatose/D-tagatose + naked HFA/D-tagatose + free DPEase/D-tagatose + HFA unblocked/D-tagatose + HFA blocked.

Free and immobilised DPEase were subjected to reactions with four different substrates, and the products were determined by HPLC analysis. The Fischer-projection formulas for the four ketohexoses are shown in Figure 7. D-fructose and D-allulose, D-tagatose and D-sorbose, and L-tagatose and L-sorbose are epimers at the C-3 position. As shown in Figure 8a,b, free and immobilised DPEase showed similar (*p* > 0.05) catalytic abilities when D-fructose and D-allulose were used as substrates. However, unlike the free enzyme, immobilised DPEase in the present study catalysed the incomplete formation of L-tagatose from L-sorbose and D-sorbose from D-tagatose (Figure 8c,d). The control group in Figure 8c shows that the L-sorbose and HFA mixture could not catalyse the formation of L-tagatose, which indicated that the change in substrate specificity of immobilised DPEase was not attributable to the effect of the resin present on it. The same was true when D-tagatose was used as a substrate (Figure 8d). Similar changes in substrate specificity after enzyme immobilisation have been reported in a few studies (e.g., pullulanase) [34].

DPEase was confirmed to be tetrameric structure [21], and the monomer structures of ketose 3-epimerases were highly similar. Each subunit is composed of eight α-helices and β-sheets, forming an evident TIM barrel structure [35]. Each monomer also contains a metal ion binding site, which is surrounded by two water molecules and four completely conserved amino acid residues (Glu, Asp, His, and Glu). The three amino acid residues (Glu, His, and Arg) that binding to O-1, O-2, and O-3 of D-fructose are also strictly conserved. In contrast, the amino acid residues that provide hydrophobic environment around O-4, O-5, and O-6 of D-fructose are markedly different. This difference in the substrate-binding pocket has been confirmed to be related to substrate-specific recognition and affinity [35,36]. This suggests that the change in substrate specificity of DPEase in our study may be attributed to the effects of immobilisation, especially covalent binding, on the conformation of enzyme (e.g., distorted substrate-binding pocket and the key amino acids involved in substrate recognition and binding).

### 3.6. Reusability

**Figure 9:**
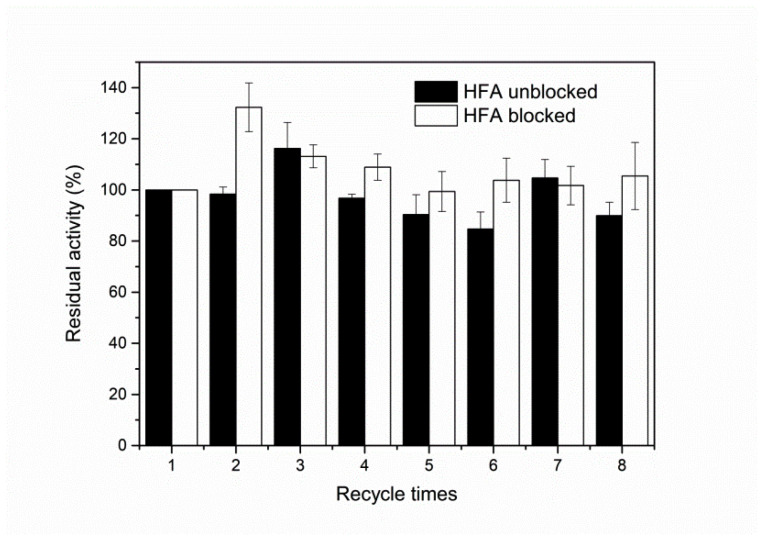
Reusability of immobilised DPEase.

The reusability of immobilised DPEase was determined by repeating reactions for eight times (Figure 9). The longer amino-epoxide spacer arms up to 27.8 Å [37] conferred flexibility to the enzyme structure [17] and lowered steric hindrance. For this reason, the active pocket was exposed for a certain duration, and a higher (*p* < 0.05) activity of immobilised enzyme was observed at a certain period. Notably, the secondary amino and thioether bonds formed by covalent binding are generally extremely stable [27]; therefore, there would be enzyme desorption from the carrier to a relatively less extent. The possible reason for the reduction in activity was the loss of resin and the inactivation of enzymes at higher temperatures for a long duration.

## 4. Conclusions

In the present study, DPEase was immobilised on an amino-epoxide support HFA with intense multipoint attachment for the first time, and its enzymatic properties were studied. The enzyme was immobilised on the support in four steps: Induction of ion exchange by secondary amine groups, covalent binding of epoxy groups to protein residues, establishment of crosslinking between glutaraldehyde and protein groups, and blocking of aldehyde groups and epoxy groups by glycine effect.

Our results showed that DPEase was successfully immobilised on HFA with the presence of excess epoxy groups that were blocked appropriately by glycine. Immobilised DPEase (blocked, unblocked) could maintain high activities over a wider range of temperature and pH. More interestingly, the immobilised enzyme could catalyse the incomplete conversion of L-sorbose to L-tagatose and from D-tagatose to D-sorbose. This may provide an alternative application of immobilised DPEase for rare sugar production. Additionally, HFA is easy to separate because of its high mechanical strength and suitable particle size. In conclusion, the results of our study may provide important insights into the production of D-allulose from D-fructose, and into the application of other rare sugars for the production of immobilised enzymes.

## Figures and Tables

**Table 1:** Immobilisation of DPEase on HFA.

Treatment	Optimum Immobilisation Condition				Immobilisation Results		
	Applied Enzyme Loads (U/g Dry Resin)	Time (h)	Temperature (°C)	pH	Enzyme Activity (U/g Dry Resin)	Activity Recovery (%)	Protein Loading Efficiency (%)
Ion exchange	200	8	20	7.5	65.6 ± 4.0 ^D^	32.8± 2.0 ^D^	58.2 ± 2.2 ^C^
Ion exchange + Covalent binding	200	8 + 12	20	7.5	89.2 ± 3.2 ^C^	44.6 ± 1.6 ^C^	77.5 ± 3.1 ^B^
Ion exchange + Covalent binding + Crosslinking	200	8 + 12 + 1	20	7.5	138.8± 2.6 ^A^	69.4 ± 1.3 ^A^	90.6 ± 2.0 ^A^
Ion exchange + Covalent binding + Crosslinking + Blocking	200	8 + 12 + 1 + 16	20	7.5	103.5 ± 2.6 ^B^	51.8 ± 1.3 ^B^	90.6 ± 2.0 ^A^

Data followed by different superscript letters on the same column denote statistically significant differences (*p* < 0.05).

## Data Availability

Data available on request due to restrictions privacy. The data presented in this study are available on request from the corresponding author. The data are not publicly available due to privacy.

## Supplementary Materials

The following are available online at https://www.mdpi.com/article/10.3390/foods10040831/s1, Figure S1: Effect of enzyme addition on activities of immobilized DPEase.
