# Supplementary figures and images for: Improved Performance of D-Psicose 3-Epimerase by Immobilisation on Amino-Epoxide Support with Intense Multipoint Attachment
Source: Foods. 2021 Apr 11;10(4):831. doi: 10.3390/foods10040831 (PMC8069956; doi:10.3390/foods10040831)

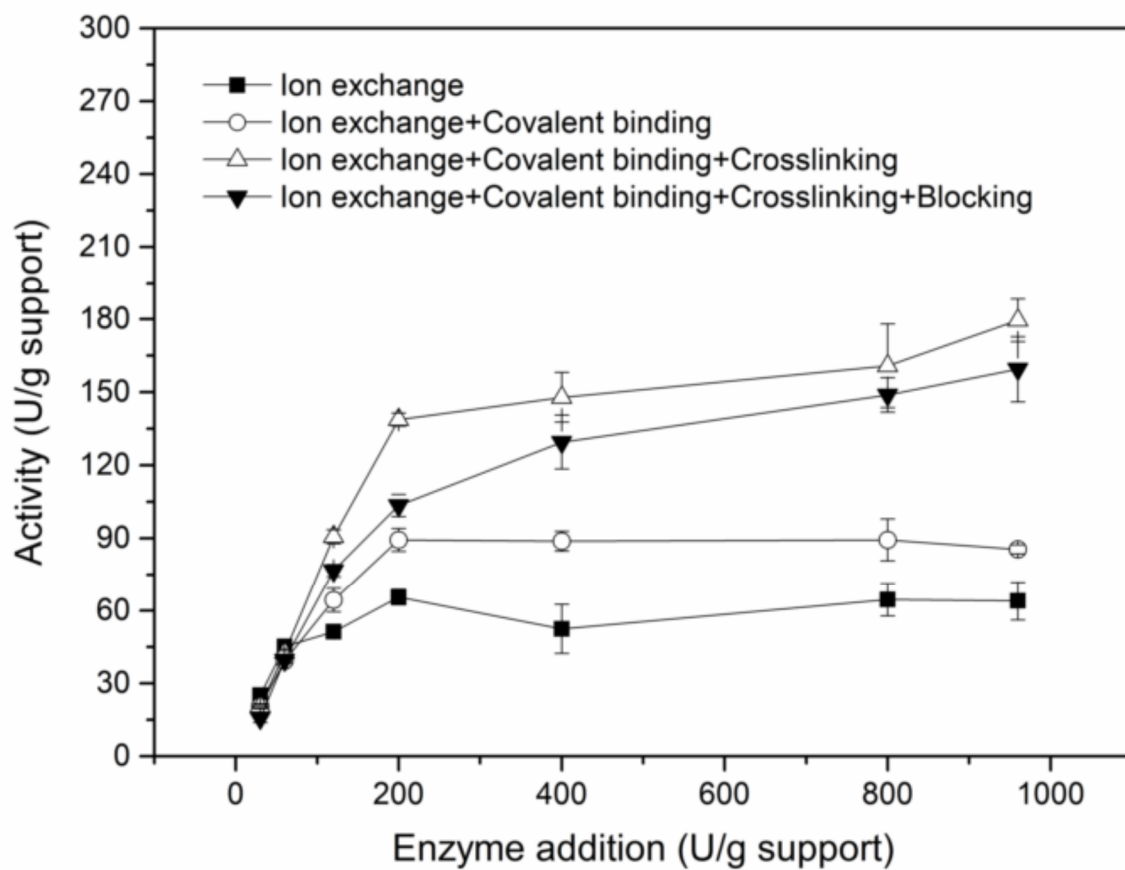

**Figure S1.** Effect of enzyme addition on activities of immobilized DPEase.

Supplement: Supplementary file 1 [file foods-10-00831-s001.pdf]
